# Spin-dependent transport properties of Fe_3_O_4_/MoS_2_/Fe_3_O_4_ junctions

**DOI:** 10.1038/srep15984

**Published:** 2015-11-02

**Authors:** Han-Chun Wu, Cormac Ó Coileáin, Mourad Abid, Ozhet Mauit, Askar Syrlybekov, Abbas Khalid, Hongjun Xu, Riley Gatensby, Jing Jing Wang, Huajun Liu, Li Yang, Georg S. Duesberg, Hong-Zhou Zhang, Mohamed Abid, Igor V. Shvets

**Affiliations:** 1 Key Laboratory of Cluster Science of Ministry of Education, School of Physics, Beijing Institute of Technology, Beijing, 100081, P.R. China.; 2 KSU-Aramco Center, King Saud University, Riyadh 11451, Saudi Arabia; 3 School of Physics and CRANN, Trinity College Dublin, Dublin 2, Ireland; 4 National Laboratory Astana, Nazarbayev University, Astana, Kazakhstan; 5 CRANN, School of Chemistry, Trinity College Dublin, Dublin 2, Ireland; 6 Institute of Plasma Physics, Chinese Academy of Sciences, Hefei, 230031, P. R. China; 7 Electronic Engineering Institute, Hefei, 230037, P. R. China

## Abstract

Magnetite is a half-metal with a high Curie temperature of 858 K, making it a promising candidate for magnetic tunnel junctions (MTJs). Yet, initial efforts to exploit its half metallic nature in Fe_3_O_4_/MgO/Fe_3_O_4_ MTJ structures have been far from promising. Finding suitable barrier layer materials, which keep the half metallic nature of Fe_3_O_4_ at the interface between Fe_3_O_4_ layers and barrier layer, is one of main challenges in this field. Two-dimensional (2D) materials may be good candidates for this purpose. Molybdenum disulfide (MoS_2_) is a transition metal dichalcogenide (TMD) semiconductor with distinctive electronic, optical, and catalytic properties. Here, we show based on the first principle calculations that Fe_3_O_4_ keeps a nearly fully spin polarized electron band at the interface between MoS_2_ and Fe_3_O_4_. We also present the first attempt to fabricate the Fe_3_O_4_/MoS_2_/Fe_3_O_4_ MTJs. A clear tunneling magnetoresistance (TMR) signal was observed below 200 K. Thus, our experimental and theoretical studies indicate that MoS_2_ can be a good barrier material for Fe_3_O_4_ based MTJs. Our calculations also indicate that junctions incorporating monolayer or bilayer MoS_2_ are metallic.

Magnetite (Fe_3_O_4_), an archetypal oxide with potential applications in spintronics, has attracted a tremendous level of attention in recent decades. It has a nearly fully spin polarized electron band at the Fermi level (half-metallic character) and a high Curie temperature of 858 K, which make it a promising candidate for room temperature spintronic devices and applications^1^,^2^,^3^. Recently, interesting spin transport properties in Fe_3_O_4_ have been reported, i.e., a spin Seebeck effect^4^, a spin filter effect^5^, an electrical field-induced phase transition^6^,^7^,^8^, large transversal magnetoresitance (MR)^9^,^10^, and charge-orbital ordering driven magnetic state switching in Fe_3_O_4_/MgO/Fe_3_O_4_ junctions^11^. Yet, initial efforts to exploit its half metallic nature in magnetic tunnel junction (MTJ) structures have been far from promising^11^. The reduced MR attained in these investigations was attributed to the formation of a dead layer at the interface between Fe_3_O_4_ layers and MgO barrier layer, which reduces the spin polarization. Finding suitable barrier layer materials is one of main challenges in this field.

Two-dimensional (2D) materials may be good candidates for this purpose. For example, graphene, an atomic-thick carbon sheet^14^,^15^, shows great potential for enhancing spin polarization. A recent experiment showed that spin dependent transport at the interfaces of Fe_3_O_4_-graphene-Fe_3_O_4_ junctions contributes −1.6% MR to the whole device^16^. This effect can be further enhanced by inserting a thin oxide layer such as TiO_2_^17^. Unlike graphene, molybdenum disulfide (MoS_2_) is a transition metal dichalcogenide semiconductor. Monolayer MoS_2_ is a direct band gap semiconductor with a band gap of 1.9 eV^18^, while bulk MoS_2_ has an indirect band gap of 1.29 eV^19^. Recently, monolayer and multilayer MoS_2_ have been shown to have a carrier mobilities of up to 200 cm^2^/Vs and 100 cm^2^/Vs at room temperature, respectively, and a current on/off ratio of 1 × 10^8^, which makes MoS_2_ a promising candidate for incorporation into a new generation of more efficient transistors^20^. Moreover, monolayer MoS_2_ has strong spin orbit coupling with a large spin splitting of up to 456 meV in the valence band originating from the d orbitals of the heavy metal atoms^21^, due to the symmetry breaking^21^,^22^. Valley polarization in monolayer MoS_2_ can be achieved with circularly polarized light^23^,^24^. Consequently, MoS_2_ is also a fascinating material for spintronics applications based on spin and valley control^25^,^26^. MoS_2_ also has potential in photoluminescence sensors^27^, solar energy funnels^28^, integrated circuit devices^29^, chemical sensor^30^, and photodiodes^31^. Recently, the magnetic and electronic properties of Fe_3_O_4_, MoS_2_ and ferromagnetic metal/Mos_2_ interfaces have been investigated^32^,^33^,^34^,^35^,^36^,^37^,^38^,^39^ and the NiFe/MoS_2_/NiFe Junction has been fabricated^40^.

In this paper, we show based on the first principle calculations that Fe_3_O_4_ keeps a nearly fully spin polarized electron band at the interface between MoS_2_ and Fe_3_O_4_ and no dead layer is formed at the interface. We also present the first report on the fabrication and characterization of MoS_2_ based MTJs with ferromagnetic oxide electrode. The MTJ stacks in this work are Fe_3_O_4_-MoS_2_-Fe_3_O_4_ trilayers grown on MgO (001) single crystal substrate. The quality of the MoS_2_ barrier layer was investigated by X-ray photoemission spectroscopy (XPS), Raman spectroscopy, atomic force microscopy (AFM), and transmission electron microscopy (TEM). The magneto-transport properties were studied by the means of a physical property measurement system (PPMS, Quantum Design). A clear tunneling magnetoresistance (TMR) signal was observed at 200 K. Our results may be also valuable for understanding how MoS_2_ can be used for other spintronic devices and applications.

## Results

### Electronic structure of Fe_3_O_4_/MoS_2_/Fe_3_O_4_ junctions

**Figure 1:**
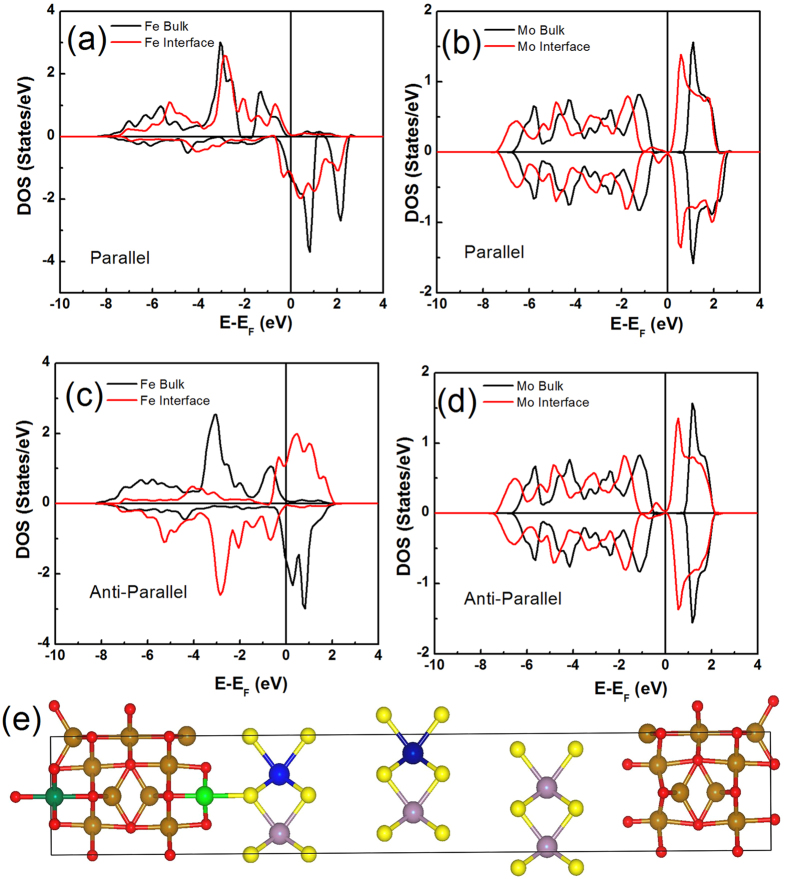
Electronic structure of Fe_3_O_4_/MoS_2_/Fe_3_O_4_ junctions. Projected density of states of Fe (dark green ball) and Mo (dark blue ball) atoms far from the interface and Fe (light green ball) and Mo (light blue ball) atoms at the interface, for magnetization of the two Fe_3_O_4_ layers in parallel (**a,b**) or in anti-parallel configurations (**c,d**). (**e**) Atomic structure of the model used for density of states calculation.

We employed the projected augmented plane wave method (PAW)^41^, implemented within the Vienna Ab Initio Simulation Package (VASP)^42^. The generalized gradient approximation^43^ is used for the exchange-correlation energy. In our calculations, we use a rotated MoS_2_ supercell constructed in a rectangular geometry and consider two reduced Fe_3_O_4_ unit cells containing 28 atoms each, separated by a 3 MoS_2_ layers, as shown in Fig. 1e. We consider MoS_2_ to be epitaxially grown on Fe_3_O_4_ as our experiment, thus the MoS_2_ is strained to match the lattice of Fe_3_O_4_. All geometries are relaxed until the forces on the atoms are less than 0.02 eV/Å. Periodic boundary conditions are employed in the plane perpendicular to the transport direction (z direction), with a monkhorst-pack 6 × 6 × 1 k-point grid for geometry optimization. In Fig. 1, we plotted the density of states (DOS) of B-site Fe (dark green ball) and Mo (dark blue ball) atoms far from the interface, and B-site Fe (light green ball) and Mo (light blue ball) atoms at the interface, for magnetization of the two Fe_3_O_4_ layers in parallel and anti-parallel configurations The MoS_2_ at the interface becomes metallic behavior due to hybridization between Fe and S, which is consistent with the reports by another research group^40^,^44^. Significantly, despite the hybridization between the Fe and S, the interface Fe still keeps a nearly fully spin polarized electron band at the Fermi level, indicating that Fe_3_O_4_ keeps its half-metallic nature in Fe_3_O_4_/MoS_2_/Fe_3_O_4_ junctions and no dead layer is formed at the interface. If we do not take into account the spin flip process when the spin polarized current tunneling through MoS_2_, the TMR of a MTJ can be defined as: 
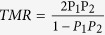
, where *P*_*1*_ and *P*_*2*_ are the spin polarization of the two ferromagnetic electrodes. Thus, a significant high TMR is expected in Fe_3_O_4_/MoS_2_/Fe_3_O_4_ junctions. In the following sections, we will describe the attempt to fabricate the Fe_3_O_4_/MoS_2_/Fe_3_O_4_ MTJs.

### Device preparation and characterization

**Figure 2:**
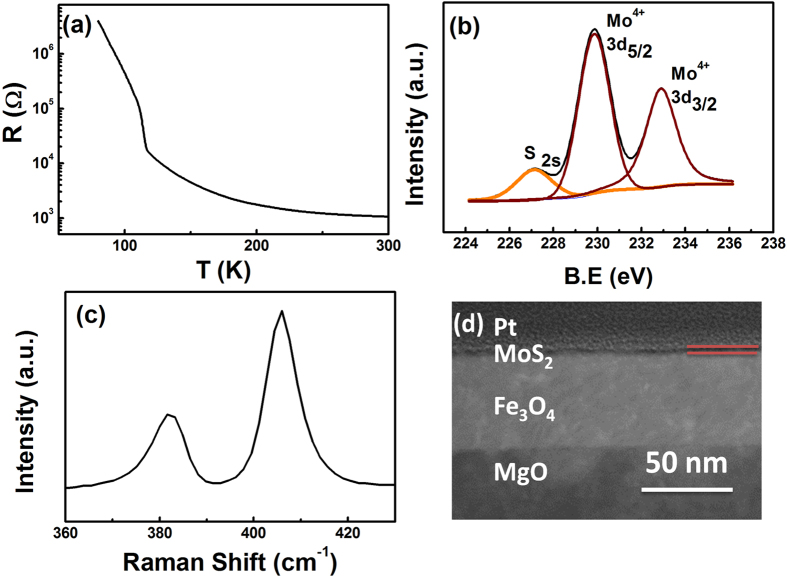
Raman and TEM characterization of Fe_3_O_4_/MoS_2_/Fe_3_O_4_ trilayer structure. (**a**) R-T curve of the bottom Fe_3_O_4_ electrode, (**b**) X-ray photoemission spectroscopy compositional analysis of the MoS_2_ layer, (**c**) Raman spectra of the MoS_2_ barrier layer, (**d**) TEM characterization of MoS_2_/Fe_3_O_4_ bilayer on a MgO substrate.

The Fe_3_O_4_/MoS_2_/Fe_3_O_4_ trilayer structures were grown on MgO (001) single crystal substrates. The thicknesses for the bottom and top Fe_3_O_4_ layers were 60 nm and 10 nm respectively. The Fe_3_O_4_ layers were prepared in a molecular beam epitaxy system (MBE) with a base pressure of 5 × 10^−10^ Torr. Reflection high energy electron diffraction (RHEED) was employed to confirm the epitaxial growth and establish the growth mode. Figure S1 shows RHEED patterns recorded in the [100] azimuth during growth, which indicates the pseudomorphic growth of the Fe_3_O_4_. Figure 2a shows the resistivity as a function of the temperature (R-T) for the bottom Fe_3_O_4_ layers. The Verwey transition is clearly present at 115 K which is close to well established bulk values, indicating the good quality of the bottom Fe_3_O_4_ layer. Details of the growth procedure and characterization are given elsewhere^45^. MoS_2_ can be obtained by the top-down exfoliation methods^46^, or by bottom up methods, such as transition metal sulfurization^47^, molybdenum oxide sulfurization^48^, decomposition of thiomolybdates^49^, and van der Waals epitaxy^50^. To fabricate few- or single-layer MoS_2_ devices, most studies use mechanical exfoliation or intercalation-assisted exfoliation of bulk MoS_2_, which results in microscale flakes of random shapes and thicknesses. In this work, to achieve large-scale continuous MoS_2_ films on the bottom Fe_3_O_4_ electrode, a thin Mo film was first deposited on the bottom Fe_3_O_4_ using an e-beam heated Mo source in the MBE system. After growth, the thin Mo layer was annealed at 500 ^°^C with an oxygen partial pressure of 1 × 10^−5^ Torr for 30 minutes. Figure S1c shows the RHEED patterns of sample after annealing in oxygen indicating the epitaxial growth of MoO on the Fe_3_O_4_ layer which also guarantees a flat interface between MoS_2_ and Fe_3_O_4_. To form MoS_2_, the sample was removed from the MBE chamber and sulfurized at 700 °C for 1 min to 2 min in a furnace based on the well-established vapor phase growth technique^51^. The chemical composition of the MoS_2_ layer was investigated by XPS (Fig. 2b). The Mo 3d_5/2_, Mo 3d_3/2_, and S 2 s peaks have been consistently energy shifted in order to position the peak in the C 1 s region at a binding energy (BE) of 284.7 eV. The peak positions for the Mo 3d_5/2_, Mo 3d_3/2_, and S 2 s are 229 eV, 232 eV, and 226 eV respectively, which are consistent with the values for bulk MoS_2_. From Fig. 2b, we can also estimate that the atomic ratio of Mo: S is approximately 1:2. Raman spectroscopy was used to further evaluate the quality of the MoS_2_ layer. Figure 2c shows a Raman spectrum of the MoS_2_ film collected by a Renishaw spectrometer with a 488 nm laser at room temperature. Strong signals for both the characteristic in-plane degenerate E^1^_2_ _g_ and out-of plane A_1_ _g_ modes are present at 406 cm^−1^ and 383 cm^−1^, respectively. The peak positions are close to the positions expected for bulk MoS_2_. The XPS and Raman results indicate the good quality of the MoS_2_ layer.

The morphology of the MoS_2_ was examined with AFM. The AFM image shown in Figure S2 indicates a uniform MoS_2_ film on the Fe_3_O_4_. Additionally, the RMS roughness of this thin film is of 0.117 nm. The quality of the MoS_2_/ Fe_3_O_4_ interface and thickness of MoS_2_ layer were characterized by TEM. Figure 2d shows a typical TEM image of the MoS_2_/ Fe_3_O_4_ bilayer on a MgO substrate. One can see from Fig. 2d that a 2 nm thick MoS_2_ film is on top of the Fe_3_O_4_ layer with a sharp interface between the MoS_2_ and Fe_3_O_4_ layers. Finally, the top Fe_3_O_4_ electrode was deposited. Two different size junctions (10 × 10 μm^2^ and 400 × 200 nm^2^) were fabricated through several steps E-beam lithography and lift-off processes as described in the experimental section.

### Magnetic and Transport properties

**Figure 3:**
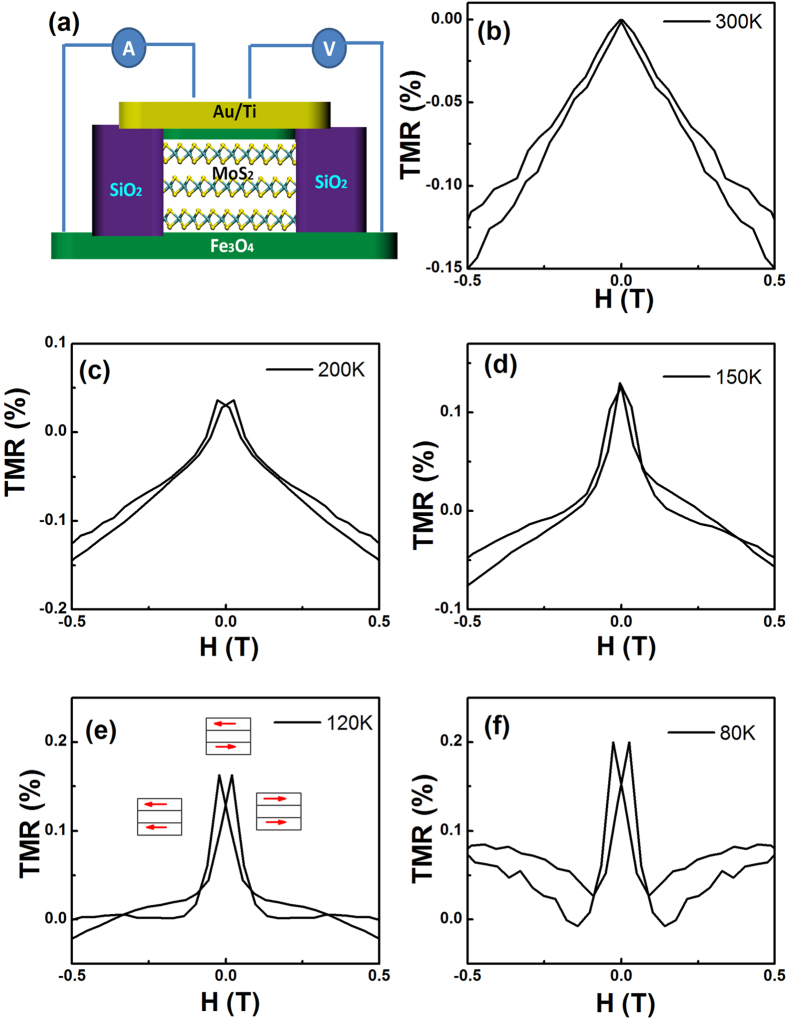
TMR of Fe_3_O_4_/MoS_2_/Fe_3_O_4_ junctions. (**a**) Schematic drawing of the Fe_3_O_4_/MoS_2_/Fe_3_O_4_ junctions. TMR curves for Fe_3_O_4_/MoS_2_/Fe_3_O_4_ junctions measured at (**b**) 300 K, (**c**) 200 K, (**d**) 150 K, (**e**) 120 K, and (**f**) 80 K respectively.

Figure 3a shows a schematic drawing of the Fe_3_O_4_/MoS_2_/Fe_3_O_4_ junctions. When a bias current is applied perpendicular to the films, a spin-polarized current is injected into the MoS_2_ barrier layer from the top Fe_3_O_4_ electrode, which can then be detected by the bottom Fe_3_O_4_ electrode. It is known that the different thicknesses of the Fe_3_O_4_ layers will result in different coercivity fields^13^. Figure S3 shows the M (H) loops for 60 nm and 10 nm of Fe_3_O_4_ on MgO substrates. The coercivity fields for 60 nm of Fe_3_O_4_ and 10 nm of Fe_3_O_4_ at room temperature are 125 Oe and 60 Oe respectively. By applying an in-plane magnetic field, the magnetization states of the device can be tuned from a parallel (P) to an antiparallel (AP) configuration, or vice versa. Figure 3b–f show MR curves of Fe_3_O_4_/MoS_2_/Fe_3_O_4_ junctions measured at different temperatures. The external magnetic field was applied in the film plane along the [100] direction. At 300 K, the resistance shows a linear response to the external field. While at low temperature, non-linear MR curves are observed. The linear MR behavior observed at room temperature is mainly due to spin-dependent transport across the anti-ferromagnetic (AF) anti-phase boundaries (APBs) in Fe_3_O_4_ layers^52^,^53^. To understand better the MR curves measured at low temperature, we show in Figure S4 the MR plots of the Fe_3_O_4_/MoS_2_/Fe_3_O_4_ junctions measured with a field of up to 1 T. One can clearly see from Figure S4 that there are two main contributions to the MR: non-linear MR at low fields and linear MR at high fields due to spin-dependent transport across the AF APBs.

**Figure 4:**
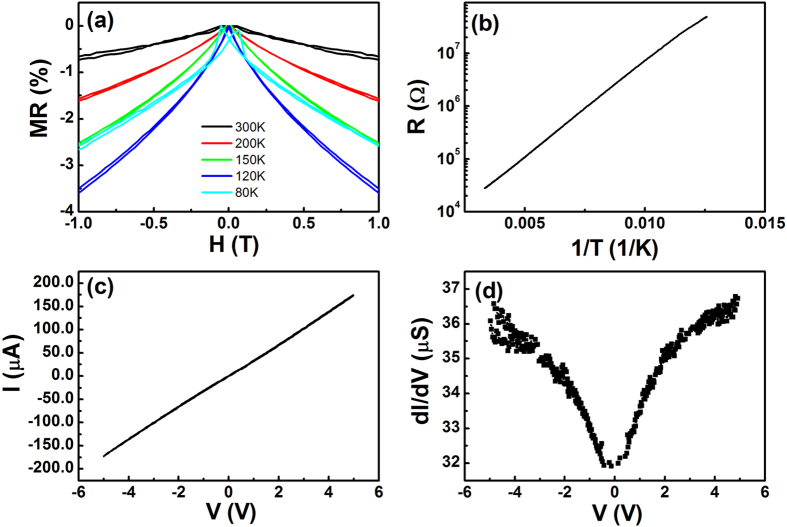
IV curve demonstrating the TMR effect of the device. (**a**) Temperature dependent MR curves for the bottom 60 nm thick Fe_3_O_4_ layer. (**b**) R-T for Fe_3_O_4_/MoS_2_/Fe_3_O_4_ junctions measured at a bias of 0.5 V. (**c**,**d**) are IV and dI/dV curves for Fe_3_O_4_/MoS_2_/Fe_3_O_4_ junctions measured at 300 K.

To demonstrate that the non-linear MR observed at low fields is not due to MR effects of the bottom or top Fe_3_O_4_ electrodes, we show in Fig. 4a MR curves for the bottom 60 nm thick Fe_3_O_4_ layer measured for different temperatures. Linear non-saturated MR curves are observed for temperatures above the Verwey temperature. Moreover, the MR ratio is significantly higher than that of the Fe_3_O_4_/MoS_2_/Fe_3_O_4_ junctions. Below the Verwey temperature, the non-linear MR at low field is due to discrete changes in the entropy and the phonon-magnon interaction^52^. We also measured the MR of Fe_3_O_4_/MoS_2_/Fe_3_O_4_ junctions without MoS_2_ barrier (Figure S5). The MR curves are very similar to that for single Fe_3_O_4_ layer. Therefore, the non-linear MR observed in the Fe_3_O_4_/MoS_2_/Fe_3_O_4_ junctions at low fields cannot be attributed to the MR effect of the bottom or top Fe_3_O_4_ electrodes. Figure 4b shows the R-T plot of the Fe_3_O_4_/MoS_2_/Fe_3_O_4_ junctions.The resistance of the MTJ is one order of magnitude higher than that of solely the bottom Fe_3_O_4_ electrode and the R-T measurement shows no clear trace of the Verwey transition indicating that the resistance of the MTJ is dominated by the properties of the MoS_2_ layer. We fitted the R-T curve with the relationship 
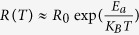
, which gives an activation energy (*E*_*a*_) of 72 meV. The obtained activation energy is about twice the value of that for the 60 nm Fe_3_O_4_ on MgO substrate and even larger than the activation energy for a single AF APB^52^. Note, while the Verwey transition is not observed in the R-T measurement, the temperature dependent magnetization (M-T) measurements still show a clear Verwey transition (Figure S6). To verify the observed non-linear MR at low fields is due to the TMR effect of the device, we show in Fig. 4c the current vs voltage (I-V) curve of the device measured at 300 K. Non-linear IV is observed. To confirm further this non-linear behavior, we show in Fig. 3d the dI/dV as a function of the bias voltage V. The quasiparabolic dependence between dI/dV and V also suggests that the non-linear MR observed at low fields is due to the TMR effect of the Fe_3_O_4_/MoS_2_/Fe_3_O_4_ junctions. We can define the TMR ratio for the low field region as 
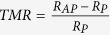
, where *R*_*AP*_ and *R*_*P*_ are resistances when the magnetization states of the device are in anti-parallel and parallel configurations respectively. Figure S7 shows the M (H) loop and MR curve for Fe_3_O_4_/MoS_2_/Fe_3_O_4_ junction measured at 200 K. To clearly see the magnetization reversal process, we have drawn schematically the magnetization states of the device in Figure S7. Therefore, the non-linear MR observed at low fields is due to the magnetization alignments of two FM electrodes. Figure S8 summarizes the TMR ratio as a function of temperature. The TMR ratio increases with decreasing temperature and a TMR ratio of 0.2% is obtained at 80 K. We did not observe a sharp increase of the TMR ratio around Verwey transition which gives another indication that the non-linear MR at low fields is due to spin-dependent tunneling through the MoS_2_ barrier layer. Although we did not obtain a TMR ratio as significant as that predicated by our calculation, our first attempt does suggest that MoS_2_ can be used as a barrier material for Fe_3_O_4_ based MTJ. One of the possible reasons for the reduced TMR ratio is that the quality of the interface between the MoS_2_ and the top Fe_3_O_4_ may be not as good as the interface between the MoS_2_ and the bottom Fe_3_O_4_ since the sample was removed from vacuum to produce the MoS_2_ layer. To confirm this, Figure S9 shows the TMR ratio as a function of bias voltage measured at 200 K. One can see that TMR is asymmetric with respect to the bias voltage and has a maximum at a bias of −1 V. This asymmetry suggests that the quality of the two interfaces is different. The bottom Fe_3_O_4_/MoS_2_ interface is atomically sharp, while the top Fe_3_O_4_/MoS_2_ interface is rougher. It also indicates that the non-linear MR observed at low fields is due to the TMR effect of the Fe_3_O_4_/MoS_2_/Fe_3_O_4_ junctions. Another possibility is that the bottom Fe_3_O_4_ may also be partially sulfurized during the sulfurization.

In conclusion, we show based on the first principle calculations and TMR characterization of Fe_3_O_4_/MoS_2_/Fe_3_O_4_ junctions that MoS_2_ can be a good barrier material for Fe_3_O_4_ based MTJs. Our calculations also indicate that junctions incorporating monolayer or bilayer MoS_2_ are metallic. Our experimental results may pave the way for the application of MoS_2_ in spintronics.

## Methods

### Growth of Fe_3_O_4_-MoS_2_-Fe_3_O_4_ trilayers

The Fe_3_O_4_-MoS_2_-Fe_3_O_4_ trilayers were grown on MgO (001) single crystal substrates using a MBE system (DCA MBE M600, Finland) with a base pressure of 5 × 10^−10^ Torr. The substrates were chemically cleaned prior to their insertion into the growth chamber and then cleaned *in situ* at 600 °C in UHV for 1 hour. The growth conditions for Fe_3_O_4_ can be found elsewhere. The thicknesses for the bottom and top Fe_3_O_4_ layers are 60 nm and 10 nm respectively. To grow MoS_2_, a thin Mo film were first deposited by e-beam from a Mo source. Subsequently, the thin Mo layer was annealed at 500 °C with an oxygen partial pressure of 1 × 10^−5^ Torr for 30 mins. Then the sample was taken out and sulfurized at 700 °C for 1 min to 2 min in a chemical vapor deposition (CVD) furnace to form MoS_2_ based on the vapor phase growth technique. This short sulfurization time prevents a deeper sulfurization of the Fe_3_O_4_ layer in agreement with the XPS analysis.

### Device fabrication

A multistep process was used to fabricate the Fe_3_O_4_-MoS_2_-Fe_3_O_4_ junctions with two different sizes (10 × 10 μm^2^ and 400 × 200 nm^2^). The resistance-area (RA) of the junction is around 10^7^ Ω μm^2^ at room temperature and remains constant irrespective to the junction size. A Negative tone S1820 and MA N2403 were used for UV lithography and e-beam lithography respectively, followed by ion milling and the post exposure lift-off was carried out after SiO_x_ deposition to insulate the top and bottom contacts. A top contact of Ti 6 nm/Au 50 nm was deposited by evaporation with subsequent lift off.

### Characterization of the Fe_3_O_4_-MoS_2_-Fe_3_O_4_ junctions

The magneto-transport behavior of the Fe_3_O_4_-MoS_2_-Fe_3_O_4_ junctions was examined by means of a physical property measurement system (PPMS, Quantum Design). X-ray photoelectron spectrometer (XPS) measurements were performed in an Omicron Nanotechnology Spectroscopy system equipped with an Ar ion miller in the preparation chamber. Raman spectra was collected using a Renishaw InVia spectrometer with a 488 nm laser at room temperature.

## Additional Information

**How to cite this article**: Wu, H.-C. *et al.* Spin-dependent transport properties of Fe_3_O_4_/MoS_2_/Fe_3_O_4_ junctions. *Sci. Rep.* **5**, 15984; doi: 10.1038/srep15984 (2015).

## Supplementary Material

Supplementary Information
