# Supplementary material for: Spin-dependent transport properties of Fe3O4/MoS2/Fe3O4 junctions
Source: Sci Rep. 2015 Nov 2;5:15984. doi: 10.1038/srep15984 (PMC4629163; doi:10.1038/srep15984)
Supplement: Supplementary Information [file srep15984-s1.pdf]

Supporting Information

Spin-dependent transport properties of Fe_3O_4/MoS_2/Fe_3O_4 junctions

Han-Chun Wu[1], Cormac Ó Coileáin[1,2,3], Mourad Abid[2], Ozhet Mauit[3], Askar Syrlybekov[3,4], Abbas Khalid[3], Hongjun Xu[3], Riley Gatensby[5], Jing Jing Wang[3], Huajun Liu[6], Li Yang[7], Georg S. Duesberg[5], Hong-Zhou Zhang[3], Mohamed Abid[2] & Igor V. Shvets[3]

[1]Key Laboratory of Cluster Science of Ministry of Education, School of Physics, Beijing Institute of Technology, Beijing 100081, People's Republic of China.

[2]KSU-Aramco Center, King Saud University, Riyadh 11451, Saudi Arabia

[3]School of Physics and CRANN, Trinity College Dublin, Dublin 2, Ireland

[4]National Laboratory Astana, Nazarbayev University, Astana, Kazakhstan

[5]CRANN, School of Chemistry, Trinity College Dublin, Dublin 2, Ireland

[6]Institute of Plasma Physics, Chinese Academy of Sciences, Hefei 230031, People's Republic of China

[7]Electronic Engineering Institute, Hefei 230037, People's Republic of China

*Correspondence and requests for materials should be addressed to H.C.W. (wuhc@bit.edu.cn) or H.J.L. (liuhj@ipp.ac.cn)

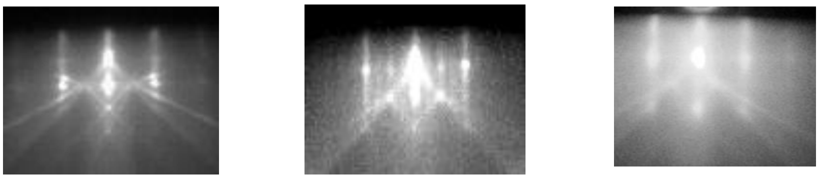

(a) **(b)** **(c)**

Figure S1. RHEED images of **(a)** UHV annealed MgO substrate, **(b)** 60 nm Fe_3O_4 (001) grown on MgO (001), and **(c)** 0.25 nm thick Mo after annealing in oxygen. The images were recorded in (100) azimuth of MgO.

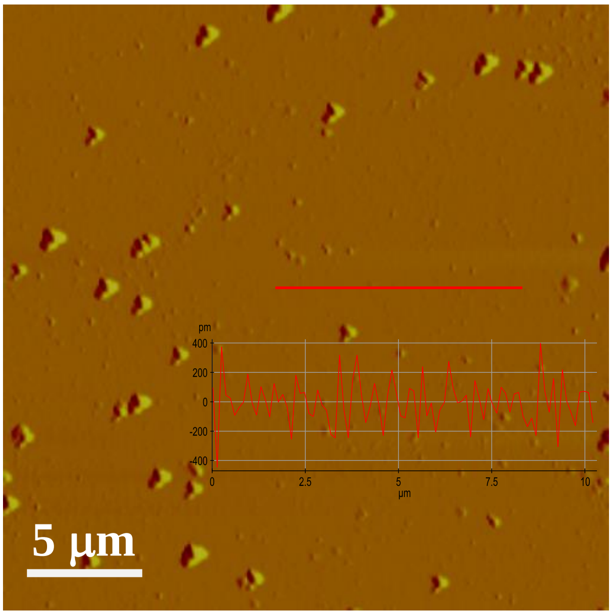

Figure S2. AFM of Fe_3O_4/MoS_2 (25 µm x 25 µm scan). Height profile along the redline is overlaid on the image and show a surface roughness of 0.12 nm. The brighter spots in the AFM picture are due to excess sulfur particles.

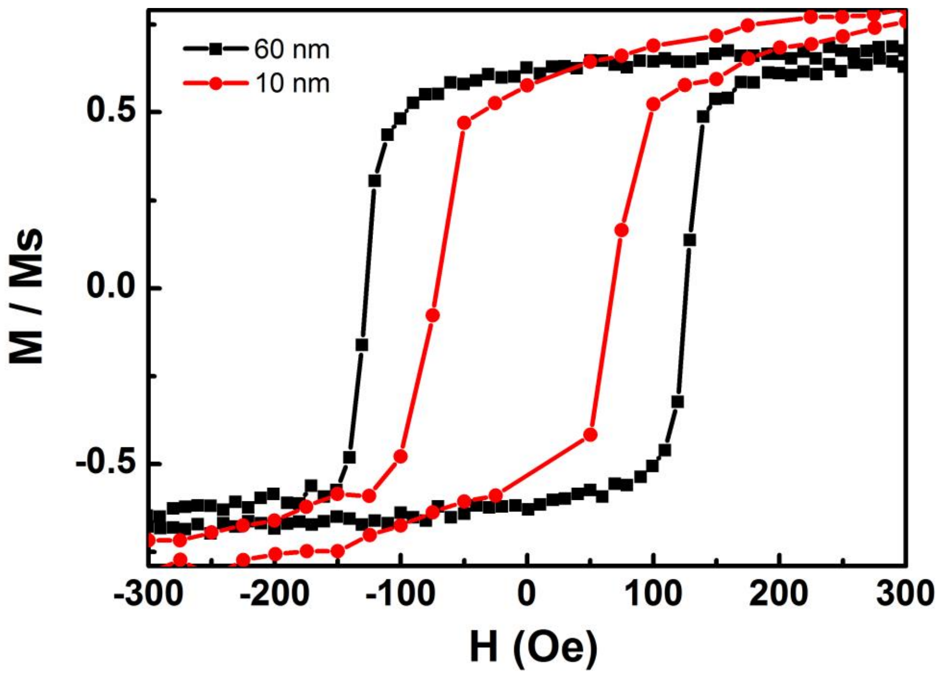

Figure S3. M (H) loops for 60 nm of Fe_3O_4 and 10 nm of Fe_3O_4 on MgO substrate measured at room temperature. The magnetic field is applied in the film plane.

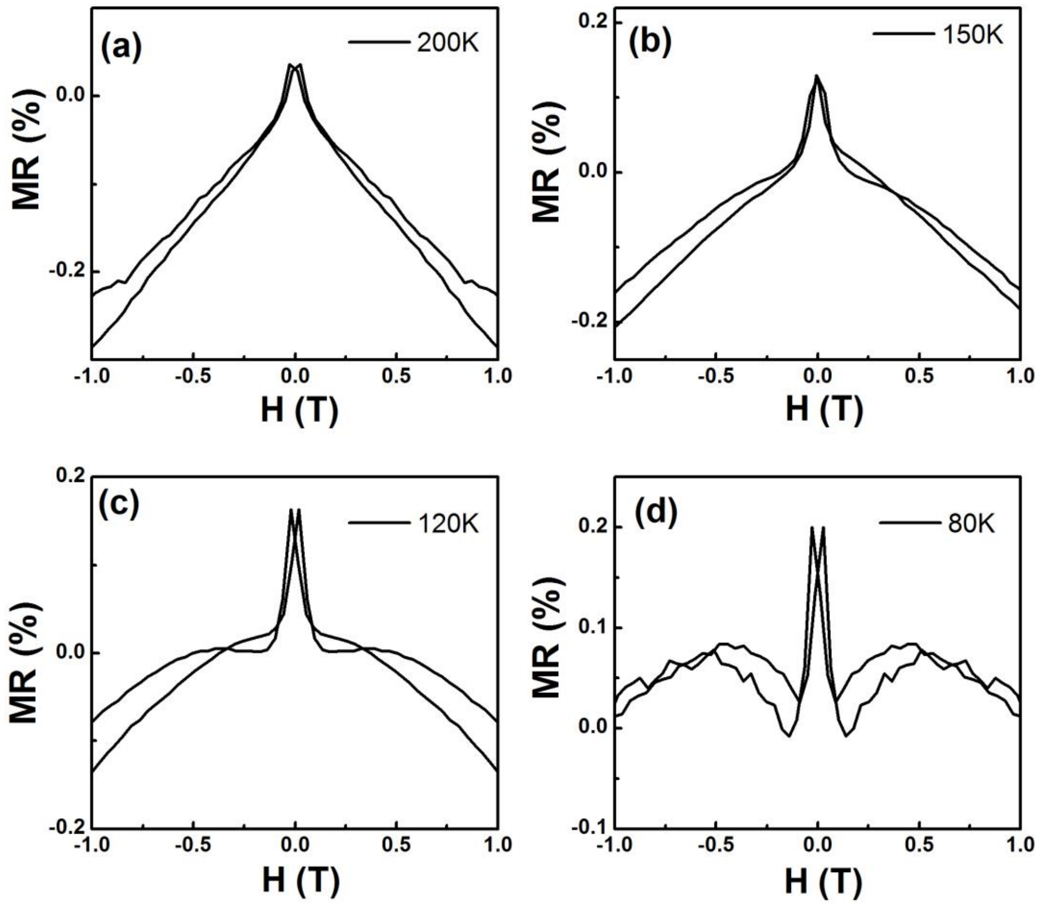

Figure S4: TMR curves for $Fe_3O_4/MoS_2/Fe_3O_4$ measured with a field of up to 1 T and at **(a)** 200 K, **(b)** 150 K, **(c)** **1**20 K, and **(d)** 80 K respectively.

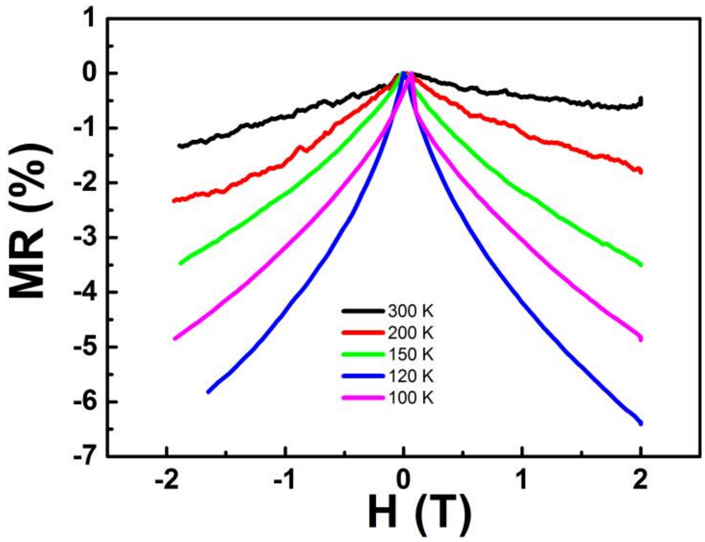

Figure S5: Temperature dependent MR curves for $Fe_3O_4/MoS_2/Fe_3O_4$ junctions without MoS_2 barrier layer.

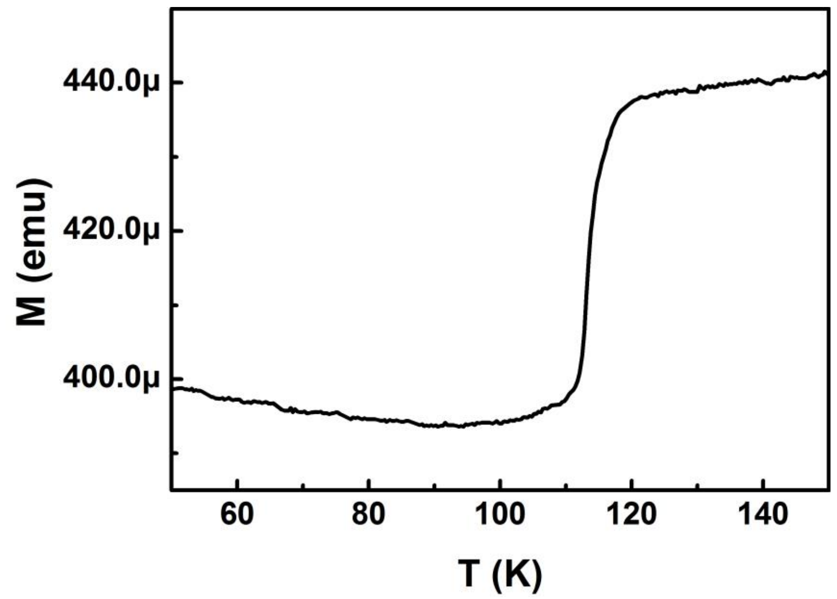

Figure S6: M-T curve for $Fe_3O_4/MoS_2/Fe_3O_4$ measured with an in-plane field of 200 Oe.

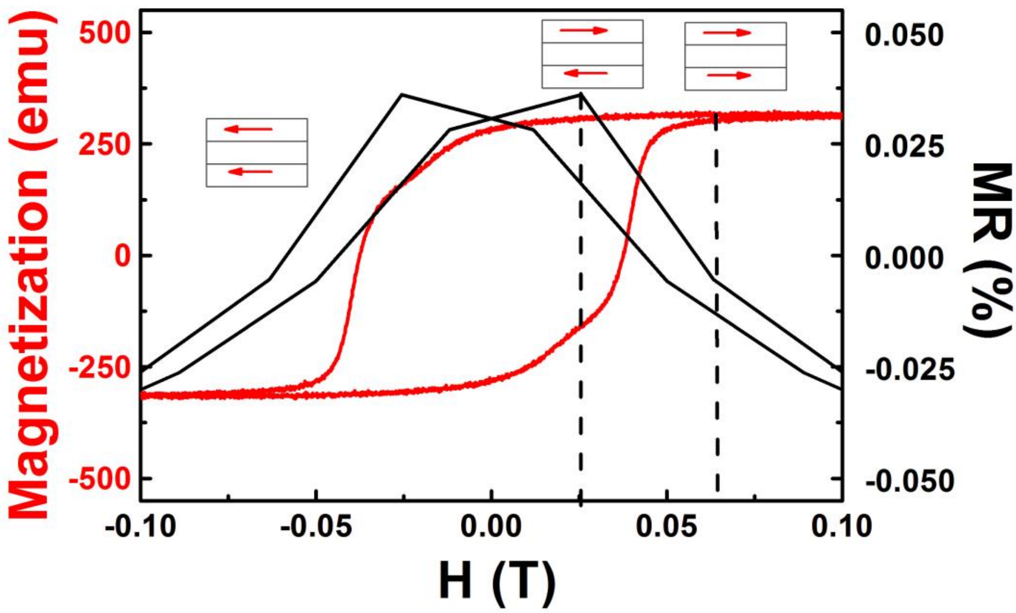

Figure S7: M (H) loop and TMR for $Fe_3O_4/MoS_2/Fe_3O_4$ junction measured at 200 K. The external magnetic field was applied in the film plane along the [100] direction.

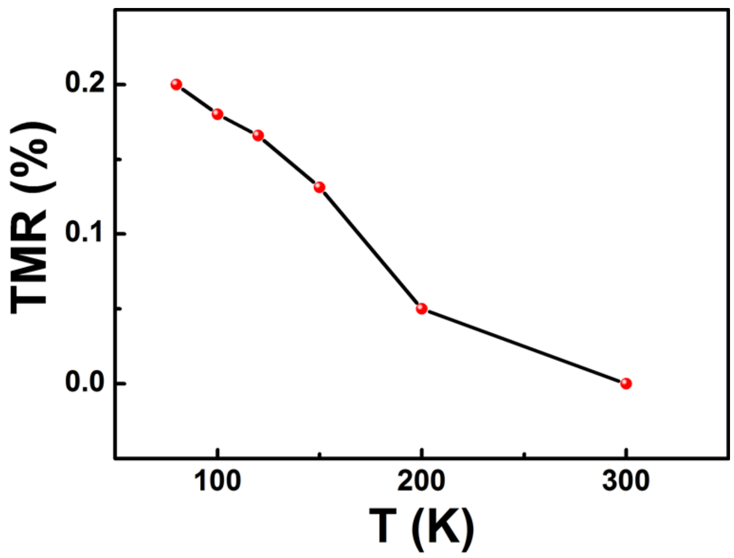

Figure S8. TMR ratio as a function of temperature for $Fe_3O_4/MoS_2/Fe_3O_4$ junctions.

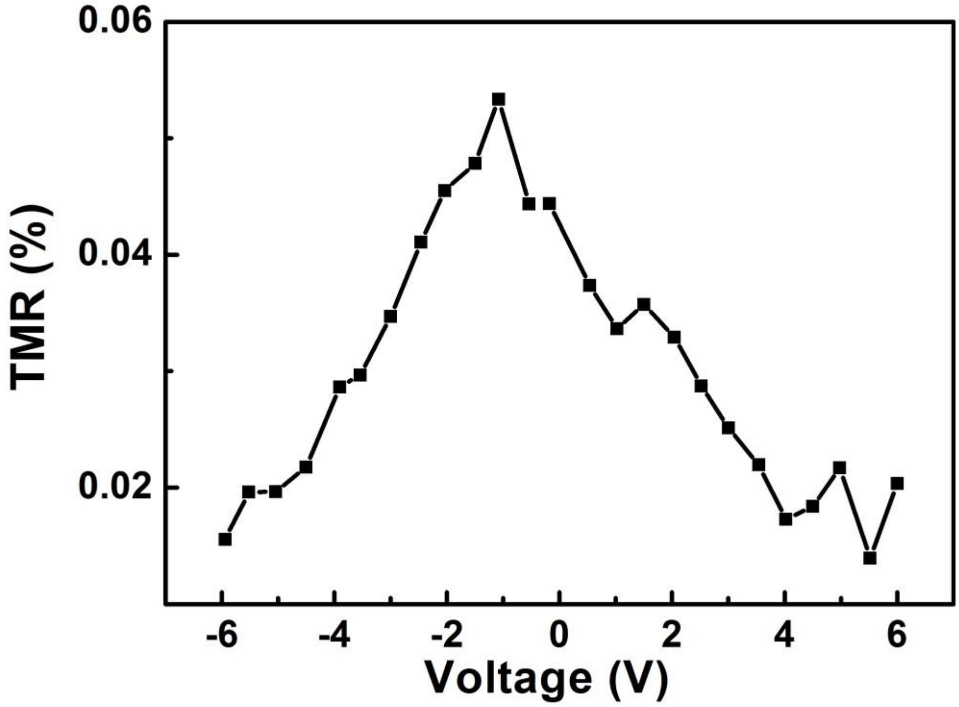

Figure S9. TMR ratio as a function of bias voltage for $Fe_3O_4/MoS_2/Fe_3O_4$ junctions measured at 200 K.
